# Regulatory Mechanism of Peroxisome Number Reduction Caused by *FgPex4* and *FgPex22-like* Deletion in *Fusarium graminearum*

**DOI:** 10.3390/jof9111083

**Published:** 2023-11-06

**Authors:** Chunjie Liu, Zhuoyu Bi, Hao Xu, Renjie Zhang, Jiayi Wang, Yuancun Liang, Li Zhang, Jinfeng Yu

**Affiliations:** Key Laboratory of Agricultural Microbiology, College of Plant Protection, Shandong Agricultural University, Tai’an 271018, China; liuchunjie2014@163.com (C.L.); 15615641621@163.com (Z.B.); xuhao5285@163.com (H.X.); 15066037747@163.com (R.Z.); 18863653601@163.com (J.W.); liangyc@sdau.edu.cn (Y.L.)

**Keywords:** peroxisome, pexophagy, peroxisome biogenesis

## Abstract

Peroxisomes are single-membrane-bound organelles that play critical roles in eukaryotic cellular functions. Peroxisome quantity is a key factor influencing the homeostasis and pathogenic processes of pathogenic fungi. The aim of the present study was to investigate the underlying mechanisms of the reduction in number of peroxisomes in *Fusarium graminearum* consequent to *FgPex4* and *FgPex22-like* deletion. The number of peroxisomes decreased by 40.55% and 39.70% when *FgPex4* and *FgPex22-like*, respectively, were absent. Peroxisome biogenesis-related proteins, as well as inheritance- and division-related dynamin-like proteins were reduced at the transcriptional level in the mutant strains. In addition, the degree of pexophagy was intensified and the accumulation of ubiquitinated FgPex5 was also increased in *F. graminearum* when *FgPex4* or *FgPex22-like* was absent. The findings suggest that *FgPex4* and *FgPex22-like* influence the number of peroxisomes by influencing peroxisome biogenesis and pexophagy.

## 1. Introduction

Peroxisomes are dynamic single-membrane organelles that are essential for the normal growth and development of eukaryotes. Peroxisomes contain at least 50 types of enzymes and participate in several biochemical pathways, such as fatty acid β-oxidation, glyoxylic acid cycle, reactive oxygen species regulation, methanol metabolism, and penicillin synthesis. Currently, more than 30 types of proteins involved in peroxisome assembly, differentiation, and genetic regulation have been reported in fungi. The proteins are called peroxins (encoding gene *Pex* and protein Pexp). Zhang et al. (2021) identified a homologous protein of Pex22p in *Fusarium graminearum* and named it FgPex22-like. The protein contains a predicted Pex4 binding site and interacts with FgPex4 as a membrane anchor protein of FgPex4 [1]. Pex4p is a ubiquitin-conjugating enzyme (UBC) and is required for efficient protein transport to peroxisomes [2,3]. FgPex4 and FgPex22-like can reportedly participate in the regulation of the development and pathogenicity of filamentous fungi [1,4].

Pex5 is a matrix protein receptor that can bind to the matrix protein containing the type 1 peroxisome target signal (PTS1) [5,6,7,8] and transport it to the lumen of peroxisomes. After unloading the matrix protein, Pex5p returns to the cytoplasm to continue its function as a receptor. The normal process of the cycle relies on the monoubiquitination of Pex5p [9,10,11,12], which is extracted from the peroxisome membrane into the cytoplasm using Pex1p and Pex6p (and also Pex15p) and initiates a new cycle following deubiquitination. Pex4p and Pex22p comprise the E2 unit required for monoubiquitination. Pex2p, Pex10p, and Pex12p play equally important roles because they are the E3 ligases of monoubiquitination of Pex5p. Once a certain peroxin related to Pex5p circulation is lost, Pex5p is unable to undergo monoubiquitination but undergoes polyubiquitination catalyzed by Ubc4p [13,14], after which it is degraded by the 26S proteasome [13].

Peroxisomes’ homeostasis is strictly regulated by their synthesis and degradation. Currently, there are two major mechanisms of peroxisome proliferation: one is de novo formation of peroxisomes, and the other is the fission of mature peroxisome. During de novo formation of peroxisomes, the endoplasmic reticulum contributes a part of the peroxisome membrane wherein the key proteins, such as Pex3p and Pex16p, are inserted to form a pre-peroxisome [15,16,17,18]. With the continuous insertion of membrane proteins and introduction of matrix proteins, peroxisomes grow and mature continuously. The continuous incorporation of the peroxisome membrane and matrix proteins during growth improves the peroxisome constantly and it matures eventually into a normally functioning organelle. The mature peroxisome can continue to extend, become tubular, and undergo cleavage to facilitate peroxisome proliferation, which is the second major pathway of peroxisome proliferation. Mature peroxisomes in *Saccharomyces cerevisiae* cells are stretched and contracted by the proteins Pex11p, Pex25p, and Pex27p and divide with the help of the dynamin-related proteins Vps1p, Dnm1p, and Fis1p [19,20,21,22,23]. After becoming an independent organelle, some peroxisomes migrate to daughter cells with the assistance of Inp1p, Myo2p, Inp2p, and Rho1p proteins [24,25,26,27,28,29,30]. Meanwhile, the remaining immature peroxisomes continue to improve by incorporating additional membrane and matrix proteins.

Autophagy is the process by which cells digest their own substances including cytoplasmic proteins and organelles. Therefore, autophagy is one of the main pathways of organelle degradation. Under nutrient-deficient, hypoxic, or damaged organelle conditions, the cell wraps some substances in the cytoplasm and transports them to lysosomes or vacuoles, wherein they are degraded to produce amino acids, monosaccharides, and other decomposition products for the cell to reuse [31,32], maintain its balance, and also help the cell to survive stress conditions [33]. Autophagy has significant implications for the growth and development of organisms, immune defense, tumor suppression, and neurodegenerative diseases. Currently, more than 40 autophagy-related (ATG) proteins are associated with autophagy in yeast [34,35,36,37], with ATG8 being the most notable. It is widely present in organisms, performs critical physiological functions, and is highly conserved across species. The ATG8 protein family participates in the formation of autophagosome membranes by binding to phosphatidylethanolamine (PE) and plays an important role in autophagosome formation [37]. Therefore, it can be used as a marker of autophagy. Pexophagy is the main pathway of peroxisome degradation [38] and is a type of selective autophagy [39,40,41]. Although the precise mechanism of pexophagy triggered by various stimuli may vary in mammals, a relatively reliable model posits that during diverse pexophagy processes, some proteins on the peroxisome membrane undergo ubiquitination in the initial stage. In addition, peroxisomal proteins play important roles in pexophagy. Macropexophagy depends on two peroxisomal proteins involved in peroxisome biogenesis. One is Pex3p, the core component of the peroxisome membrane protein (PMP)-targeting mechanism, which must be removed from the membrane and subjected to proteasome treatment [42]. The second is Pex14p, a docking protein in the peroxisomal protein import machinery. Pex14p is necessary for macropexophagy because it can act as a docking protein for the initial factor of pexophagy [43,44]. Pex1p, Pex6p, Pex15p, and Pex13p also play critical roles in pexophagy regulation [45,46].

The peroxisome number is strictly regulated by their biosynthesis and pexophagy. A study has shown that the during the *F. graminearum* infection process, the number of peroxisomes increases significantly [47]. In addition, a decrease in protein expression levels or coding gene deletion of Pex1p, Pex2p, Pex4p, Pex11p, and Pex14p can lead to a decrease in the number of peroxisomes in fungi [4,48,49,50,51,52]. Considering current knowledge on peroxisome synthesis and degradation in various organisms, the aim of the present study was to investigate the regulatory mechanisms underlying the reduction in the number of peroxisomes in *FgPex4* and *FgPex22-like* gene knockout strains of *F. graminearum.* Peroxisome origin (biogenesis, inheritance, and division of peroxisomes) and degradation (autophagy and pexophagy) were analyzed. According to the results of the present study, the reduction in the number of peroxisomes caused by the deletion of *FgPex4* and *FgPex22-like* genes is a consequence of both the blockage of the source pathway and the intensification of peroxisome autophagy.

## 2. Materials and Methods

### 2.1. Fungal Strains and Growth Assays

The transformants used in the present study were obtained by protoplast transformation of the wild-type strain PH-1 of *F. graminearum*. Double-knockout mutants were generated using the split-marker approach [53,54] based on single-knockout mutants. Single knockout mutants were obtained using hygromycin genes, whereas the double knockout mutants were obtained using *G418* genes. The single knockout mutants, Δ*FgPex4* and Δ*FgPex22-like*, have been described previously [1,4]. The strains were cultured using different media depending on the objective of the experiment. The well-known potato dextrose agar (PDA) (200 g potato, 15–20 g glucose, 15 g agar, and 1 L water) medium was used to transfer the cultures of the strains.

### 2.2. Strain Construction

*FgPex4* and *FgPex22-like* genes were knocked out in the laboratory prior and maintained in sterile water at 4 °C. The primers used to amplify each target gene are listed in Table S1. The open reading frame of each targeted gene was replaced with a geneticin (*G418*) resistance cassette. *ATG1* (FGSG_05547) was knocked out in Δ*FgPex4* to establish a double-knockout mutant strain ΔΔ*ATG1/FgPex4* using geneticin (G418) as a screening marker. A double-knockout mutant strain ΔΔ*ATG1/FgPex22-like* was constructed using the same method. All the mutants were identified via PCR using specific primers (Table S1).

The native promoter, GFP sequence, and coding sequence of ATG8 (FGSG_10740) of *F. graminearum* were amplified using primers ATG8-1-F/R, ATG8-2-F/R, and ATG8-3-F/R, respectively, and then linked by overlapping PCR (three pairs of primers, Table S1). The purified DNA product was linked to *Xho*I-digested *pFL2* vector using a cloning enzyme and then introduced into *Escherichia coli* strain DH5α to propagate the recombined *pFL2-GFP-ATG8* plasmid. Other recombinant plasmids *pFL2-Pex14-GFP*, *pFL2-Pex5-GFP*, *pFL2-mCherry-PTS1*, and *pFL2-PTS2-GFP* were constructed using the same strategy. The FGSG_10362 hypothetical protein, which is predicted to encode the long-chain-fatty-acid CoA ligase and contains the conserved PTS1 signal peptide (AKL) at the C-terminus, was selected as a peroxisomal matrix protein PTS1 marker in the present study. Similarly, the FGSG_04243 3-ketoacyl-CoA thiolase, which is found in the National Center for Biotechnology Information database and retains a typical PTS2 signal peptide, was used as a peroxisomal matrix protein PTS2 marker. According to the different experimental purposes, the specially constructed recombinant vector was transformed into wild-type strains and deletion mutants using previously described methods [55]. *E. coli* containing recombinant plasmid was stored in 15% glycerol at −20 °C. All the fungal transformants were conserved in sterile water at 4 °C.

### 2.3. Statistics of the Number of Peroxisomes

The strain was cultured in CM medium for 24 h, and then the mycelia were taken to the center of the slide to make a temporary slide specimen. The number of peroxisomes in the mycelia was observed using a laser confocal microscope, the LSM800 (Zeiss Laboratories, Gottingen, Germany). An area 150 µm away from the top of the mycelium was selected for observation, with each sample observed to be approximately 1000 µm in length.

### 2.4. Quantitative Real-Time PCR

Total RNA was isolated from hyphae of the PH-1, Δ*FgPex4,* and Δ*FgPex22-like* strains using RNA isolator (Total RNA Extraction Reagent, Vazyme Biotech Co., Ltd., Nanjing, China). cDNA was reverse-transcribed from an equivalent amount of RNA. qRT-PCR experiments were performed according to the manufacturer instructions for Hieff^®^ qPCR SYBR^®^ Green Master Mix, No Rox (Yeasen Biotechnology Co., Ltd., Shanghai, China) containing DNA polymerase, SYBR Green I, dNTPs, and Mg^2+^. qRT-PCR was performed by simply adding templates and primers to the amplification system, greatly simplifying the operation process and reducing the probability of contamination. A Lightcycler^®^ 96 instrument (Roche Inc., Branchburg, NJ, USA) was used for DNA strand amplification, signal detection, and data display. The quantitative results were only analyzed when the amplification curve was S-shaped, the Ct value fell between 20 and 30, and the melting curve was a single peak. The relative expression level of each gene was calculated using a previously described method [56].

### 2.5. Autophagy and Pexophagy Detection

The recombinant plasmid *pFL2-GFP-ATG8* was introduced into the wild-type strain PH-1 and mutant strains Δ*FgPex4* and Δ*FgPex22-like* via PEG-mediated transformation. The correct transformant strains PH-1/GFP:FgATG8, Δ*FgPex4*/GFP:FgATG8, and Δ*FgPex22-like*/GFP:FgATG8 were cultured in liquid complete medium (CM) (10 g dextrose, 2 g tryptone, 1 g yeast extract, 1 g casamino acids, 6 g NaNO_3_, 0.5 g KCl, 1 g MgSO_4_·7H_2_O, 1.5 g KH_2_PO_4_, 15 g agar, 1 L water, pH 6.5) at 25 °C for 24 h, and the mycelia were transferred to liquid nitrogen-deficient medium MM-N (1 g KH_2_PO_4_, 0.5 g KCl, 0.5 g MgSO_4_·7H_2_O, 0.01 g FeSO_4_·7H_2_O, 30 g sucrose, 1 L water, pH 6.9, added another 200 μL fusarium trace elements: 5 g citric acid, 5 g ZnSO_4_·7H_2_O, 0.25 g CuSO_4_·5H_2_O, 100 mL water) for another 12 h. Mycelia cultured in the above different media were stained with 10 µM CMAC (7-amino-4-chloromethylcoumarin) solution, and their autophagy was observed under a Zeiss LSM800 laser confocal microscope (Zeiss Laboratories, Gottingen, Germany). Mycelial samples were obtained for Western blotting analysis using the same culture method. The total proteins were extracted with filamentous fungal protein extraction kit (BestBio, Shanghai, China) and their concentrations were determined using the bicinchoninic acid method, separated with 12.5% sodium dodecyl sulfate-polyacrylamide gel electrophoresis (SDS-PAGE), transferred to 0.45 µm polyvinylidenefluoride membrane (Millipore, Schwalbach, Germany), and hybridized using GFP monoclonal antibody and β-actin monoclonal antibodies. β-actin was used as a loading control. The assay method of pexophagy was similar to that of autophagy. The difference was that *pFL2-Pex14-GFP* was introduced into three different strains instead of *pFL2-GFP-ATG8*, correct transformant strains were transferred to oleate medium (1% peptone, 0.3% yeast extract, 0.1% oleate, and 1% Tween-20) for 20 h after being cultured in CM for 24 h, and were then transferred to MM-N medium for 12 h. Mycelial samples were analyzed using SDS-PAGE and immunoblotting. Autophagy and pexophagy under different conditions were assessed based on the proportion of free GFP in protein immunoblotting.

### 2.6. Polyubiquitination Detection of Pex5

Strains PH-1, Δ*FgPex4*, and Δ*FgPex22-like* were cultured in potato dextrose broth (PDB, liquid PDA) medium at 25 °C and 180 rpm for 1 d, filtered with sterile three-layer lens paper, rinsed with pre-cooled PBS buffer, quickly dried, and frozen in liquid nitrogen. The subsequent experiments (protein extraction, trypsin digestion, affinity enrichment, LC-MS/MS analysis) and database search were performed as described previously [57,58]. In addition, ubiquitination of Pex5p (FGSG_01174) detected via Co-IP*. pFL2-Pex5-GFP* carrier was introduced into the protoplasts of PH-1, Δ*FgPex4*, and Δ*FgPex22-like*, and the transformants were verified using PCR. The total proteins (Input) were also extracted using a filamentous fungal protein extraction kit (BestBio, Shanghai, China) and their concentrations were determined using the bicinchoninic acid method. An equivalent amount of protein was detected using Western blot analysis with an anti-GFP (1:5000 dilution, Abcam, Cambridge, UK) and anti-actin (1:2000 dilution, Huadingbio, Nanjing, China) antibody. Western blots of proteins eluted from the protein A/G-agarose-anti-GFP were detected with the anti-GFP and anti-ubiquitin (1:1000 dilution, Cell Signaling Technology, Boston, MA, USA) antibody. IgG served as a negative control to exclude the possibility of contamination in the experiment.

### 2.7. Detection of the Recovery of Peroxisome Function

The strains were inoculated onto a solid oleic acid medium (6 g sucrose was replaced by 160 μL oleic acid and 5 mL NP40 in 200 mL MM) with oleic acid as the sole carbon source. Inoculated Petri dishes were inverted and incubated at 25 °C for 7 days. Growth was observed and images were recorded. In addition, the strains were inoculated into a liquid oleic acid medium and shaken for 7 days at 25 °C and 180 rpm. The mycelia were collected and placed in an oven until the weight of the mycelia remained constant. Finally, the dry weight of the mycelium was confirmed. The experiment was repeated multiple times.

### 2.8. Determination of Matrix Protein Introduction

The constructed vectors *pFL2-mCherry-PTS1* and *pFL2-PTS2-GFP* were transformed into strains PH-1, Δ*FgPex4*, and Δ*FgPex22-like* using protoplast transformation methods, and the correct transformants were obtained via PCR verification. The correct transformants were inoculated in 100 mL liquid CM, cultured at 25 °C and 180 rpm for 1 day. The mycelia were picked onto a glass slide, and their fluorescence was observed using a laser confocal microscope, the LSM800.

## 3. Results

### 3.1. Deletion of FgPex4 and FgPex22-like Genes Reduced Number of Peroxisomes

**Figure 1:**
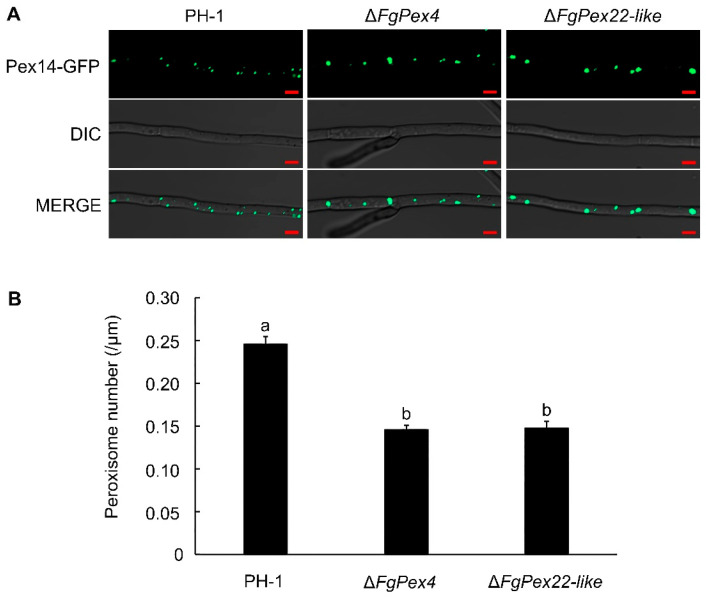
Effect of *FgPex4* and *FgPex22-like* gene deletion on the number of peroxisomes. (**A**) Peroxisomes in mycelia. Several mycelia from the edge of the colony were transferred to 100 mL CM medium and cultured for 24 h in an incubator at 25 °C (180 rpm shaking speed). Fluorescence of young mycelia was observed using an LSM800 laser confocal microscope. Scale bar = 5 μm. (**B**) Number of peroxisomes per unit length of mycelia. We manually counted the number of peroxisomes in approximately 1000 μm of mycelium. Different letters on the bars for each treatment indicate significant difference at *p* < 0.05 by Duncan’s multiple range test. Measurements represent the average of three independent experiments. Error bars on the histograms represent the standard error of three repeated tests.

To investigate the effect of *FgPex4* and *FgPex22-like* gene knockout on the number of peroxisomes in *F. graminearum*, green fluorescent protein (GFP) was used to label the peroxisomal membrane protein FgPex14 in PH-1, Δ*FgPex4*, and Δ*FgPex22-like* strains, and the number of fluorescent dots in the mycelia cultured in liquid complete medium (CM) for 24 h was subsequently observed with an LSM800 laser confocal microscope to estimate the number of peroxisomes in each strain. For the main observation area, a region 150 μm away from the top of the mycelium was selected. Each strain was observed with a mycelium length of approximately 1000 μm. The results showed that the number of peroxisomes in *F. graminearum* decreased by 40.55% and 39.70% after the deletion of *FgPex4* and *FgPex22-like* genes, respectively (Figure 1).

### 3.2. Peroxisome Synthesis Affected by the Knockout of FgPex4 and FgPex22-like Genes

**Figure 2:**
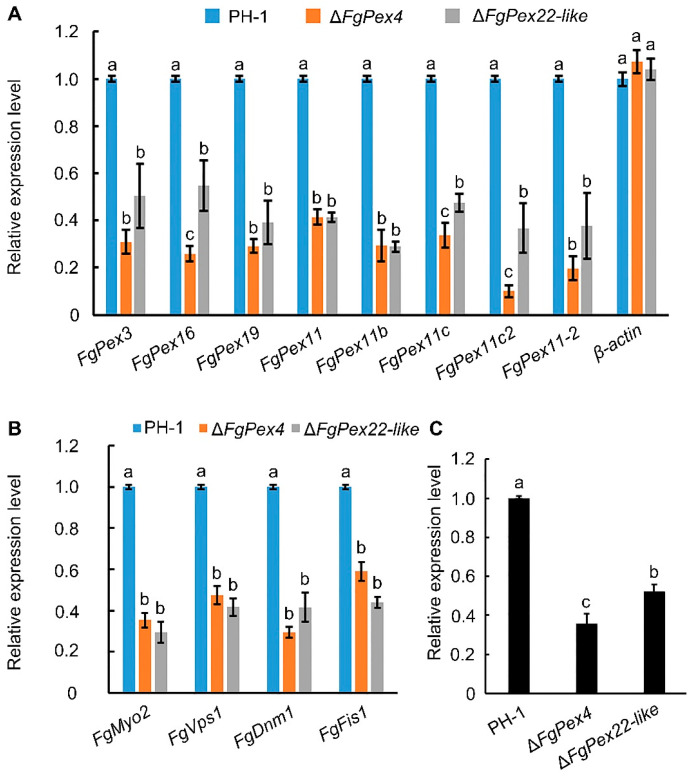
Effect of *FgPex4* and *FgPex22-like* deletion on peroxisome synthesis pathway of *Fusarium graminearum*. (**A**) Relative quantitative analysis of peroxisome synthesis-related genes at the transcriptional level. The mycelia were collected and cultured in liquid CM medium for 24 h, and RNA was extracted and reverse-transcribed for repeated qRT-PCR experiments. GAPDH was used as an internal reference gene. (**B**) Relative quantitative analysis of peroxisome inheritance and division-related genes at transcriptional level. The total RNA of three strains (wild-type PH-1, Δ*FgPex4*, and Δ*FgPex22-like*) was obtained using the same method, and then a series of experiments and analyses were performed. GAPDH was used as an internal reference gene. (**C**) Relative quantitative analysis of PMP70 at the transcriptional level. Error bars on the histograms represent the standard error of three repeated tests. Different letters on the bars for each treatment indicate significant difference at *p* < 0.05 by Duncan’s multiple range test. All experiments were repeated three times with three replicates each time.

To investigate the impact of *FgPex4* and *FgPex22-like* deletions on peroxisome synthesis, the expression at the transcriptional level of proteins related to synthesis, division, and inheritance was examined. Fresh hyphae of the strains under study were transferred into 100 mL of CM liquid medium and cultured at 25 °C and 180 rpm. After 24 h of culture, the hyphae were collected to extract RNA and reverse-transcribed to obtain cDNA for use in subsequent experiments. Differences in the expression of peroxisome synthesis-related genes, *FgPex3*, *FgPex16*, *FgPex19*, *FgPex11*, *FgPex11b*, *FgPex11c*, *FgPex11c2*, *FgPex11-2*, and dynamic genes related to inheritance and division, *FgMyo2*, *FgVps1*, *FgDnm1*, and *FgFis1*, were detected at the transcription level using quantitative real-time PCR (qRT-PCR). Simultaneously, a difference in the expression of *PMP70* at the transcriptional level, which accounts for a large proportion of PMPs, was detected. The internal reference used in this experiment was glyceraldehyde 3-phosphate dehydrogenase (GAPDH). The results showed that in both the *FgPex4* and *FgPex22-like* gene knockout strains, the expression of peroxisome biogenesis-related genes, inheritance- and division-related genes, and the *PMP70* gene at the transcriptional level was significantly lower than that of the wild-type strain PH-1 (Figure 2). β-actin, a gene with little relationship with peroxisome, was used as an additional reference gene. Therefore, after the deletion of *FgPex4* and *FgPex22-like* genes, one of the factors that decreased the number of peroxisomes was that the source of peroxisomes was affected.

### 3.3. Knockout of FgPex4 and FgPex22-like Genes Affected Peroxisome Degradation

#### 3.3.1. Effect of *FgPex4* and *FgPex22-like* Deletions on Autophagy

**Figure 3:**
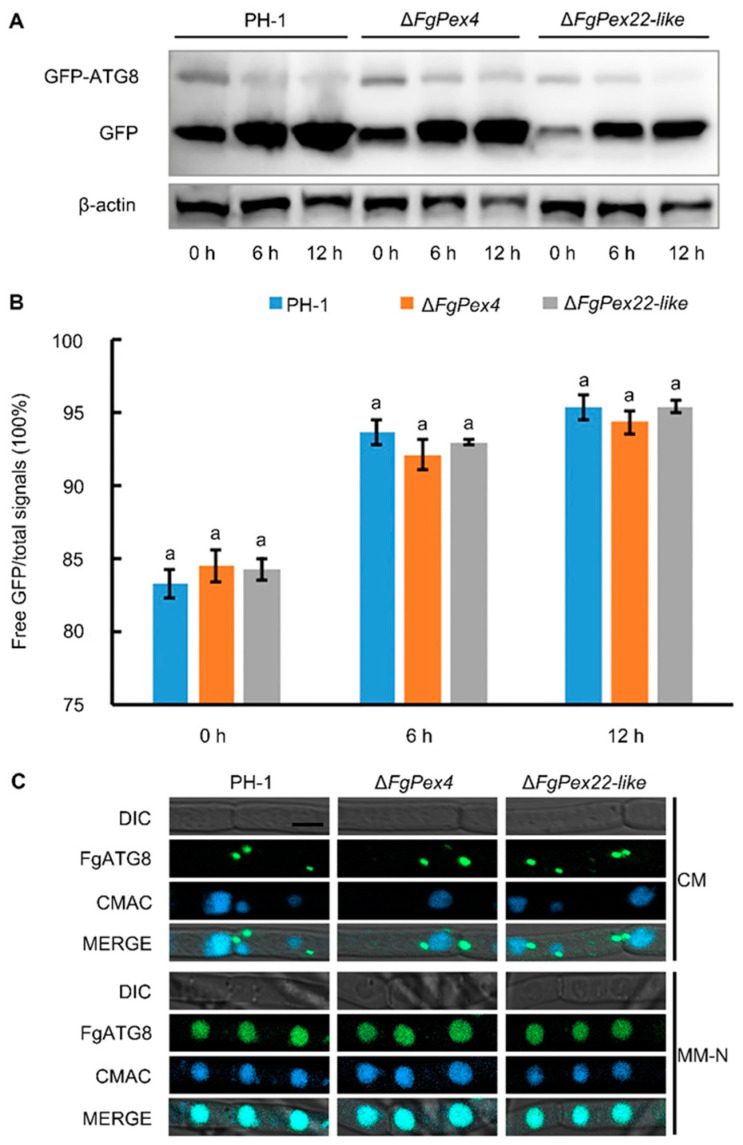
Effect of *FgPex4* and *FgPex22-like* deletion on autophagy. (**A**) Western blotting was used to detect the autophagy of the strains induced in nitrogen-deficient medium MM-N for 0, 6, and 12 h. The fresh mycelia at the edge of the fungal colony were transferred into CM medium and incubated for 24 h and then transferred to MM-N medium for 0, 6, and 12 h. The results of anti-GFP antibody detection were used to identify autophagy of the strain. β-actin was used as an internal control. (**B**) The calculated proportion of free GFP in the total band signal. Error bars on the histograms represent the standard error of three repeated tests. Different letters on the bars for each treatment indicate significant difference at *p* < 0.05 based on Duncan’s multiple range test. (**C**) Detection of autophagy fluorescence signal. Strains PH-1/GFP:FgATG8, Δ*FgPex4*/GFP:FgATG8, and Δ*FgPex22-like*/GFP:FgATG8 were transferred from the CM culture medium to the MM-N culture medium for 12 h, stained with 10 µM CMAC, and the distribution of fluorescence in the mycelia observed under a laser confocal microscope. Scale bar = 10 µm. Green fluorescence represents GFP-FgATG8 protein, blue fluorescence represents vacuoles stained by CMAC, and turquoise represents their colocalization.

Autophagy-related degradation pathways were investigated to verify if the reduction in the number of peroxisomes caused by knockout of *FgPex4* and *FgPex22-like* genes was related to difference in degree of peroxisome degradation. Autophagy has become a popular research topic in recent decades. In-depth research has revealed proteins that can be used to label autophagosomes, with the autophagy-related protein ATG8 being the most recognized. Free GFP indicates that GFP-ATG8 is degraded during autophagy, so that the higher the proportion of free GFP in the total signal in each lane, the greater the degree of autophagy. The vector *pFL2-GFP-ATG8* was introduced into the protoplast of PH-1, Δ*FgPex4*, and Δ*FgPex22-like* strains to obtain the correct transformants PH-1/GFP:FgATG8, Δ*FgPex4*/GFP:FgATG8, and Δ*FgPex22-like*/GFP:FgATG8. Mycelia cultured in CM medium for 24 h were transferred to nitrogen-deficient medium (MM-N) and collected after 0, 6, and 12 h. Total protein was extracted from the hyphal samples collected at different intervals and detected with an anti-GFP antibody. The proportion of free GFP in the total signal was calculated according to the gray value of the band. β-actin was used as an internal parameter. The results showed that the degree of autophagy of Δ*FgPex4* and Δ*FgPex22-like* was not significantly different from that of the wild type strain PH-1 cultured in CM for 24 h and nitrogen-deficient medium (MM-N) for 0, 6, and 12 h (Figure 3A,B and Figure S1). The hyphae were stained with 10 µM 7-amino-4-chloromethylcoumarin (CMAC) to track the location of the vacuoles, and blue fluorescence in the vacuoles and green fluorescence of the GFP:FgATG8 protein were observed using laser confocal microscopy. Regardless of Δ*FgPex4* or Δ*FgPex22-like*, the mycelia cultured in CM were similar to the wild-type strain PH-1. GFP:FgATG8 was either located at the phagophore assembly site next to the vacuole or diffused in the cytoplasm. After MM-N-induced autophagy, the three strains presented the same state; that is, GFP:FgATG8 was located in the vacuole, and autophagy occurred (Figure 3C). This indicates that autophagy was not associated with the decrease in the number of peroxisomes in the Δ*FgPex4* and Δ*FgPex22-like* strains.

#### 3.3.2. Deletion of *FgPex4* and *FgPex22-like* Aggravated the Pexophagy of *F. graminearum*

**Figure 4:**
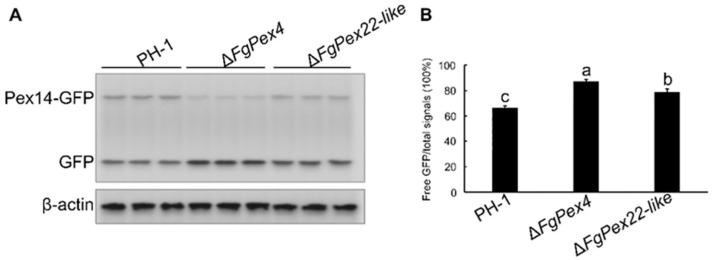
Pexophagy in Δ*FgPex4* and Δ*FgPex22-like*. (**A**) After the mycelia were cultured in MM-N medium for 24 h, Pex14:GFP proteolysis was examined using Western blot in PH-1/FgPex14:GFP, Δ*FgPex4*/FgPex14:GFP, and Δ*FgPex22-like*/FgPex14:GFP strains. (**B**) Statistics of the degree of Pex14:GFP protein hydrolysis. Gray value of the strip was estimated and the proportion of GFP in the total signal was calculated using ImageJ software (1.53a). Error bars on the histograms represent the standard error of three repeated tests. Different letters on the bars for each treatment indicate significant difference at *p* < 0.05 based on Duncan’s multiple range test.

To clearly determine whether pexophagy was related to the decrease of peroxisome number, the *pFL2-FgPex14-GFP* vector was introduced into PH-1, Δ*FgPex4*, and Δ*FgPex22-like* protoplasts to obtain the correct transformants PH-1/FgPex14:GFP, Δ*FgPex4*/FgPex14:GFP, and Δ*FgPex22-like*/FgPex14:GFP. The total hyphal protein was extracted, and anti-GFP antibody was used to detect FgPex14-GFP protein and free GFP protein in the total protein; pexophagy was preliminarily predicted according to the proportion of free GFP after inducing nitrogen deficiency for 0 and 24 h. Anti-β-actin antibody was used as internal reference antibody. The results indicated that the color of free GFP bands in strains Δ*FgPex4* and Δ*FgPex22-like* was darker, and the value of the ratio of free GFP to total signals was also higher compared to the wildtype PH-1. This observation was consistent whether the mycelia were cultured in CM for 24 h or induced by nitrogen deficiency for 24 h (Figure 4 and Figure S2). The above results indicate that the loss of *FgPex4* and *FgPex22-like* could aggravate pexophagy. Therefore, it can be inferred that the decrease in the number of peroxisomes in Δ*FgPex4* and Δ*FgPex22-like* strains was also associated with pexophagy.

### 3.4. Effect of FgPex4 and FgPex22-like Genes Deletion on Polyubiquitination of Pex5p

**Figure 5:**
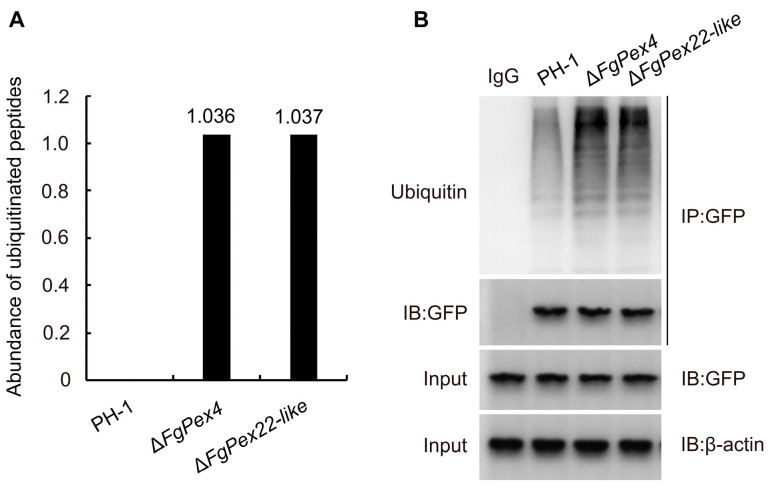
Effect of the deletion of *FgPex4* and *FgPex22-like* on ubiquitinated FgPex5 accumulation. (**A**) Quantitative proteomics study of ubiquitination modification of FgPex5. The protein mixtures extracted from mycelial samples of three different strains were enzymatically hydrolyzed with trypsin and analyzed using liquid chromatography-mass spectrometry. (**B**) CO-IP results of FgPex5 ubiquitination accumulation. FgPex5 is labeled with GFP. The extracted protein mixtures and proteins co-purified with FgPex5-GFP from these protein mixtures (GFP IP) were detected with the anti-GFP antibody, and then the ubiquitination of FgPex5 protein was detected with an anti-ubiquitin antibody. Anti-actin antibody was used as an antibody for the control.

The signal of peptide ubiquitination can be measured using label-free quantification and date-dependent acquisition methods. The results showed that when Pex5p could normally function as a peroxisome target signal receptor in wild-type PH-1, no specific ubiquitination modified signals with lysine as a ubiquitination site were detected. However, specific signals of ubiquitination modification sites were detected in Δ*FgPex4* and Δ*FgPex22-like* strains (Figure 5A). The constructed *pFL2-FgPex5-GFP* vector was introduced into the protoplasts of wild-type strain PH-1 and mutant strains Δ*FgPex4*, Δ*FgPex22-like* via protoplast transformation, and the protein was extracted from the correct transformants. The protein mixture and FgPex5-GFP protein co-purified from the protein mixture were detected using a GFP antibody, and the ubiquitination of FgPex5 in the sample was detected using a ubiquitin antibody. The results showed that compared with the wild-type PH-1, ubiquitination of FgPex5 increased significantly in the mutants Δ*FgPex4* and Δ*FgPex22-like* (Figure 5B). The result highlights that ubiquitinated Pex5p accumulation in the two mutant strains was significantly stronger than that in the wild-type strain.

### 3.5. Effect of Blocking the Pexophagy Pathway on the Number of Peroxisomes

**Figure 6:**
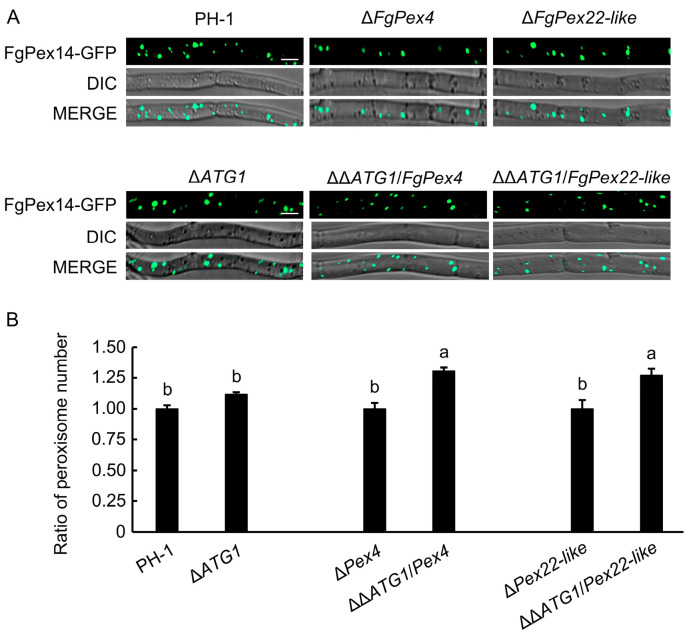
Effect of blocking the autophagy pathway on the number of peroxisomes. (**A**) Peroxisomes in mycelia. *ATG1* was knocked out in the background of PH-1/FgPex14:GFP, Δ*FgPex4*/FgPex14:GFP, and Δ*FgPex22-like*/FgPex14:GFP strains to obtain the double knockout mutants Δ*ATG1*/FgPex14:GFP, ΔΔ*ATG1/FgPex4*/FgPex14:GFP, and ΔΔ*ATG1/FgPex22-like*/FgPex14:GFP. The six strains were cultured in liquid PDB medium for 24 h, and the distribution of peroxisomes was observed under a laser confocal microscope. Scale bar = 5 μm. Green represents the peroxisome labeled with GFP, while turquoise represents the peroxisome with green fluorescence in the bright field. (**B**) Trends in the number of peroxisomes. The ratio of peroxisome number of Δ*ATG1*/PH-1, ΔΔ*ATG1/FgPex4*/Δ*FgPex4*, and ΔΔ*ATG1/FgPex22-like*/Δ*FgPex22-like* was analyzed. Error bars on the histograms represent the standard error of three repeated tests. Different letters on the bars for each treatment indicate significant difference at *p* < 0.05 based on the Duncan’s multiple range test.

A decrease in the number of peroxisomes in Δ*FgPex4* and Δ*FgPex22-like* strains aggravated pexophagy. Therefore, whether the number of peroxisomes increase in the mutant strains if pexophagy-related pathways in organisms are blocked remained unclear. Split-marker PCR technology was used to construct an ATG1 knockout fragment, which was introduced into the PH-1/FgPex14:GFP, Δ*FgPex4*/FgPex14:GFP, and Δ*FgPex22-like*/FgPex14:GFP strains via protoplast transformation. After PCR validation, the correct mutants Δ*ATG1*, ΔΔ*ATG1/Pex4*, and ΔΔ*ATG1/Pex22-like* were obtained. The number of peroxisomes in the six strains was determined using laser confocal microscopy. The results showed that the number of peroxisomes in ΔΔ*ATG1/Pex4*, and ΔΔ*ATG1/Pex22-like* mutants was higher than that in Δ*FgPex4*/FgPex14:GFP, and Δ*FgPex22-like*/FgPex14:GFP strains (Figure 6). This result demonstrated that the decrease in the number of peroxisomes in Δ*FgPex4* and Δ*FgPex22-like* mutant strains was related to pexophagy.

### 3.6. Effects of FgPex4 and FgPex22-like Deletions on Peroxisome Integrity

**Figure 7:**
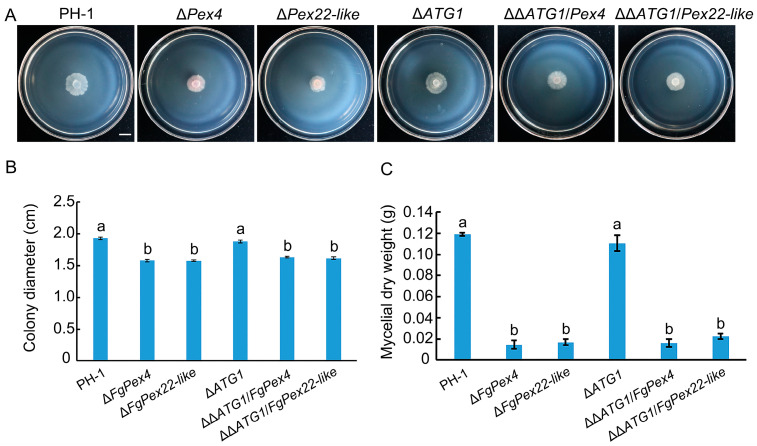
Effect of blocking pexophagy on fatty acid utilization. (**A**) Growth of each strain on oleic acid medium at 25 °C for 7 days. Scale bar = 1000 μm (**B**) Diameter measurement of each strain. (**C**) Dry weight of mycelium of each strain in liquid oleic acid medium at 7 days post-incubation (25 °C, 180 rpm). Error bars on the histograms represent the standard error of three repeated tests. Different letters on the bars for each treatment indicate significant difference at *p* < 0.05 by Duncan’s multiple range test. Measurements represent the average of three independent experiments.

**Figure 8:**
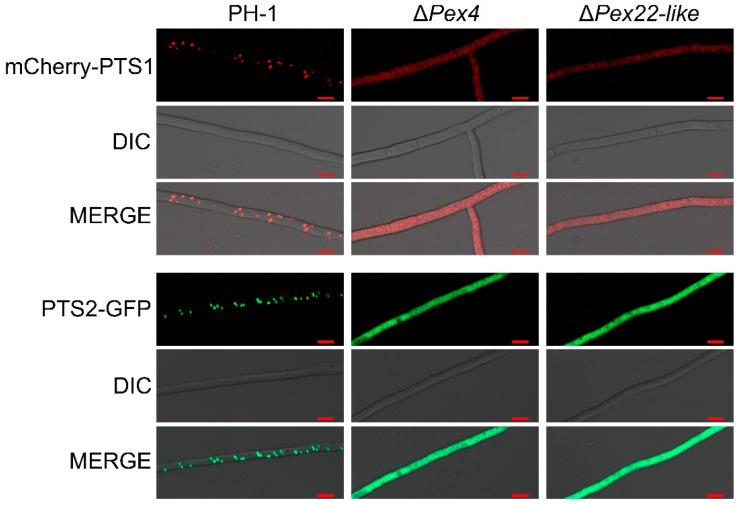
Effect of deletion of *FgPex4* and *FgPex22-like* on the introduction of peroxisome matrix protein. The strains containing mCherry:PTS1 or PTS2:GFP were cultured in liquid CM for 1 day, and then the fluorescence distribution in the mycelia was observed using a laser confocal microscope LSM800. Scale bar = 10 µm. Red fluorescence and green fluorescence represent the distribution status of mCherry:PTS1 and PTS2:GFP in the mycelia, respectively.

As damage to peroxisomes can also cause pexophagy, we examined whether peroxisomal function was damaged. The colony diameters of wild-type PH-1, two gene-deficient strains Δ*FgPex4* and Δ*FgPex22-like*, and three *ATG1* deficient strains, Δ*ATG1*, ΔΔ*ATG1/FgPex4*, and ΔΔ*ATG1/FgPex22-like*, growing on oleic acid medium, were examined. The results showed that ΔΔ*ATG1/FgPex4* and ΔΔ*ATG1/FgPex22-like* did not return to normal growth after blocking pexophagy (Figure 7A,B). Furthermore, the dry weight of the mycelia was determined, which showed no significant difference in the mycelium quantity between Δ*ATG1* and wild-type strains, whereas the mycelial dry weights of ΔΔ*ATG1/FgPex4* and ΔΔ*ATG1/FgPex22-like* did not revert to the wild-type level (Figure 7C). Labeling PTS1 and PTS2 with mCherry and GFP, respectively, showed that the loss of *FgPex4* and *FgPex22-like* prevented matrix proteins from entering the peroxisomes normally (Figure 8). This resulted in damaged peroxisomes, which may also be a reason for the intensification of pexophagy.

## 4. Discussion

In eukaryotes, peroxisomes are dynamic organelles that respond rapidly to various stimuli. In the filamentous fungus *Neurospora crassa*, the deletion of the *Pex33* not only affects the biogenesis of glyoxysomes and Woronin bodies but also reduces the density of peroxisomes [59]. In *S. cerevisiae*, Pex30p, Pex31p, and Pex32p, along with the upstream Pex28p and Pex29p*,* regulate the proliferation of peroxisomes, and deletion of *Pex30* could increase the number of peroxisomes [60]. Pex11p is a key protein in peroxisomal division. With the cooperation of the Pex25p and Pex27p membrane proteins, Pex11p is believed to be involved in the elongation and constriction process that occurs during peroxisome fission. Our results indicated that FgPex4 and FgPex22-like are also closely related to the regulation of peroxisome quantity, and the deletion of *FgPex4* and *FgPex22-like* resulted in a reduction in peroxisome quantity in Δ*FgPex4* and Δ*FgPex22-like* strains (Figure 1). In summary, FgPex4 and FgPex22-like are two essential peroxins that maintain the dynamic balance of peroxisomes in *F. graminearum*.

During peroxisome inheritance and division, several dynamin-like proteins, such as Vps1p, Dnm1p, and Dnm1p-anchoring protein Fis1p [19,20,21,22], participate in the mitochondrial division process. The proteins related to peroxisome synthesis, heredity, and division were downregulated at the transcriptional level, and the main components of the peroxisome membrane, PMP70, were also downregulated in Δ*FgPex4* and Δ*FgPex22-like* strains (Figure 2). Myo2p is a V-type myosin that translocates peroxisomes into dividing cells in *S. cerevisiae* [19,28]. Although there is no direct evidence in filamentous fungi that Myo2p is associated with peroxisome transport, our results do indicate that the absence of peroxins FgPex4 and FgPex22-like seriously affects the expression of Myo2p at the transcriptional level. Furthermore, in the NCBI database, comparisons of Myo2p between *S. cerevisiae* and *F. graminearum* showed higher sequence consistency, and their structural domains were also very similar. Therefore, we speculate that Myo2p in *F. graminearum* is also related to peroxisome transport. Based on the results, it is evident that the deletion of *FgPex4* and *FgPex22-like* affects the normal regulation of these proteins at the transcriptional level, thus affecting the expression of proteins and normal proliferation of peroxisomes.

Pexophagy is the main pathway of peroxisome degradation, and Pex14p is a key protein in pexophagy that determines whether pexophagy can occur [40,44,61]. Therefore, the degree of degradation of Pex14-GFP protein can indicate the degree of pexophagy. In the present study, the deletion of *FgPex4* and *FgPex22-like* accelerated the degradation of Pex14-GFP (Figure 4), thereby indicating the intensification of pexophagy. In *Hansenula polymorpha*, Pex4p are not required for this pexophagy [61]. Therefore, we speculate that the role of Pex4p orthologs is likely to not be conserved in fungal pexophagy.

In mammals, ubiquitination of peroxins, such as Pex5p, Pex3p, and PMP34, is believed to be involved in the activation of pexophagy [62,63,64]. Some studies have shown that in human cells, the accumulation of ubiquitinated Pex5p on the peroxisome membrane is considered a signal of pexophagy through the combination of the ubiquitin-binding protein NBR1 [65], suggesting that the accumulation of polyubiquitinated receptors may be a signal for pexophagy in mammalian cells [62]. To verify pexophagy in *F. gramiearum* is associated with the accumulation of ubiquitinated Pex5p, we detected ubiquitinated Pex5p accumulation and observed that the absence of *FgPex4* and *FgPex22-like* did indeed cause ubiquitinated Pex5p accumulation. Similarly, previous studies have found that the absence of pex1p, Pex13p, and Pex26p can lead to the accumulation of ubiquitinated Pex5p and induces pexophagy [46,66]. Therefore, the accumulation of ubiquitinated Pex5p may be closely related to pexophagy in filamentous fungi.

After the main degradation pathway of peroxisomes was blocked, the number of peroxisomes increased significantly; however, their function did not recover (Figure 7). In addition, the deletion of *FgPex4* and *FgPex22-like* not only caused the mislocalization of matrix protein with PTS1 signal but also caused the mislocalization of matrix protein with PTS2 signal (Figure 8), a result consistent with the defect trait caused by the deletion of *Pex4* and *Pex22* in the fungus *Podospora anserina* [3]. Therefore, we speculate that the reason for the lack of restoration of peroxisome function is due to peroxisome dysfunction.

In summary, the decrease in the number of peroxisomes caused by the absence of *FgPex4* and *FgPex22-like* is due to the inhibition of peroxisome synthesis and the intensification of pexophagy. Moreover, the absence of *FgPex4* and *FgPex22-like* can lead to the accumulation of ubiquitinated Pex5p and the mislocalization of matrix proteins with PTS1 or PTS2 signals. In future studies, we will try to explore the causes of pexophagy associated with *FgPex4* and *FgPex22-like* deletions.

## Data Availability

Not applicable.

## Supplementary Materials

The following supporting information can be downloaded at: https://www.mdpi.com/article/10.3390/jof9111083/s1, Table S1. Primers used in this study. Figure S1. Autophagy detection of Δ*FgPex4* and Δ*FgPex22-like* strains cultured in CM for 24 h. (A) Western blotting was used to detect the autophagy of the strains cultured in CM for 24 h. The fresh mycelia at the edge of the fungal colony were transferred into CM medium and incubated for 24 h. The results of anti-GFP antibody detection were used to identify autophagy of the strain. β-actin was used as an internal control. (B) The calculated proportion of free GFP in the total band signal. Error bars on the histograms represent the standard error of three repeated tests. Different letters on the bars for each treatment indicate significant difference at *p* < 0.05 based on Duncan’s multiple range test. Figure S2. Pexophagy detection of Δ*FgPex4* and Δ*FgPex22-like* strains cultured in CM for 24 h. (A) WB was used to detect the degree of pexophagy in strains PH-1/FgPex14:GFP, Δ*FgPex4*/FgPex14:GFP, and Δ*FgPex22-like*/FgPex14:GFP. Mycelia were cultured in CM medium for 24 h. Anti-GFP antibody was used to detect the degree of degradation of Pex14-GFP and β-actin was used as an internal control. (B) The calculated proportion of free GFP in the total band signal. The grayscale value of the strip was measured using ImageJ software. Error bars on the histograms represent the standard error of three repeated tests. Different letters on the bars for each treatment indicate significant difference at *p* < 0.05 based on Duncan’s multiple range test.
