# Supplementary material for: Regulatory Mechanism of Peroxisome Number Reduction Caused by FgPex4 and FgPex22-like Deletion in Fusarium graminearum
Source: J Fungi (Basel). 2023 Nov 6;9(11):1083. doi: 10.3390/jof9111083 (PMC10672079; doi:10.3390/jof9111083)
Supplement: Supplementary file 1 [file jof-09-01083-s001.zip › jof-2695162-supplementary.pdf]

Table S1. Primers used in this study

Primers	Sequences （5’-3’）	Relevant characteristics
FgPex14-GFP-F	caccatcaccatcactcgagGTTCTGACGAAAGGAACACTATGC	Vector construction of *pFL2-Pex14-GFP*
FgPex14-GFP-R	cgcccttgctcacctcgagAGAGCTTGAACTTGCGCCAG	
FgPex5-GFP-F	caccatcaccatcactcgagTCTTGGCAATTTGTGGTATTGG	Vector construction of *pFL2-Pex5-GFP*
FgPex5-GFP-R	cgcccttgctcacctcgagAAACTCAAACTCTGGTCTGAACACG	
ATG1-A-F	TTAGTCACCTGCCCTTGTCCG	A and B fragments for knocking out *ATG1*
ATG1-A-R	ccaaaataagcattgatgtgttgacctcccccAGCTTCCTGTTTGTTGTTATCGATG	
ATG1-B-F	aacaatacttaaataaatactactcagtaataacAGCCCAGGAGGCGGATTT	
ATG1-B-R	GCGTCCTGGCGAGCAAGT	
Nat1-F	GGGGGAGGTCAACACATCAATG	the front of Nourseothricin fragment
Nat1-R	GTCGTACAGGGCGGTGTCC	
Nat2-F	CCTGACCAAGGTGTTCCCC	the later of Nourseothricin fragment
Nat2-R	GTTATTACTGAGTAGTATTTATTTAAGTATTGTTTG	
FgATG1-F	TTCCCAAGTCACTCAGGCAA	*FgATG1* fragment
FgATG1-R	TCGGCAAATCCATAAACACA	
ATG1-K1-F	CGTAAACGCCTGAGATTGAATCT	ATG1-K1 fragment
ATG1-K1-R	TGAATCCAGAAAAGCGGCC	
ATG1-K2-F	GATCGAATTCATGGGTACCACTC	ATG1-K2 fragment
ATG1-K2-R	CACGAACTCTGCCTCTGCCT	
ATG8-1-F	CGACTCACTATAGGGCGAATTGGGTACTCAAATTGGGAACAACTGAGAACTCGGGTGA	amplify GFP-*FgATG8* sequence
ATG8-1-R	GGTGAACAGCTCCTCGCCCTTGCTCACCATGTTGA	

	CGGTGATGGTTGTTG	
ATG8-2-F	ACCATCACCGTCAACATGGTGAGCAAGGGCGAGG AGCTGTT	amplify GFP-*FgATG8* sequence
ATG8-2-R	CCTTGAATTTGCTGCGCTTGTACAGCTCGTCCATGC CGAGAG	
ATG8-3-F	CTCGGCATGGACGAGCTGTACAAGCGCAGCAAATT CAAGGACGA	amplify GFP-*FgATG8* sequence
ATG8-3-R	CTTTATAATCACCGTCATGGTCTTTGTAGTATACGCC AACGGGTCCTTTCGC	
PTS1-1-F	tcaccatcaccatcactcgagGATGGAAAGTTACTAAGTGTTA GTTAGACCA	Vector construction of *pFL2-mCherry-PTS1*
PTS1-1-R	cgcccttgctcaacatTTTGTGATTATTGATTATCGGTTAGAT G	
PTS1-2-F	caaaATGTTGAGCAAGGGCGAGG	
PTS1-2-R	gaagggtagaCTTGTACAGCTCGTCCATGCC	
PTS1-3-F	gctgtacaagTCTACCCTTCAATCCGCAATCA	
PTS1-3-R	ctcgcccttgctcacctcgagTTACAGCTTGGCCTTGGGAG	
PTS2-F	tcaccatcaccatcactcgagGTTTGTGGAGTCCGCTTCCA	Vector construction
PTS2-R	ctcgcccttgctcacctcgagGACCTGCTCGTTCACGAACAG	of *pFL2-PTS2-GFP*

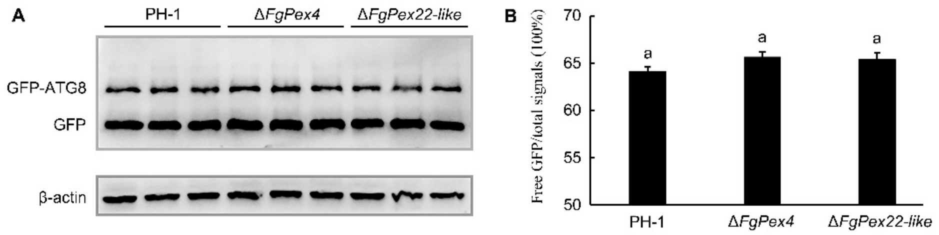

Supplementary Figure S1. Autophagy detection of Δ*FgPex4* and Δ*FgPex22-like* strains cultured in CM for 24 h. (A) Western blotting was used to detect the autophagy of the strains cultured in CM for 24 h. The fresh mycelia at the edge of the fungal colony were transferred into CM medium and incubated for 24 h. The results of anti-GFP antibody detection were used to identify autophagy of the strain. β-actin was used as an internal control. (B) The calculated proportion of free GFP in the total band signal. Error bars on the histograms represent the standard error of three repeated tests. Different letters on the bars for each treatment indicate significant difference at $p < 0.05$ based on Duncan's multiple range test.

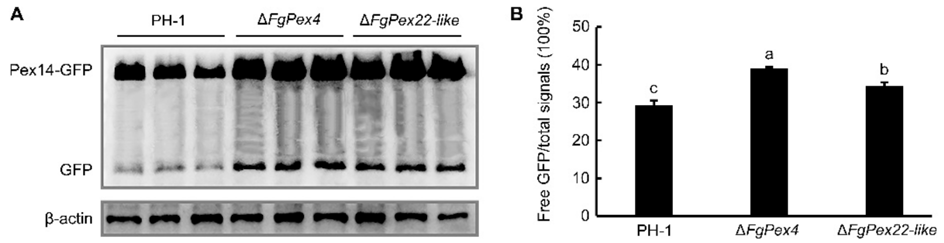

Supplementary Figure S2. Pexophagy detection of Δ*FgPex4* and Δ*FgPex22-like* strains cultured in CM for 24 h. (A) WB was used to detect the degree of pexophagy in strains PH-1/FgPex14:GFP, Δ*FgPex4*/FgPex14:GFP, and Δ*FgPex22-like*/FgPex14:GFP. Mycelia were cultured in CM medium for 24 h. Anti-GFP antibody was used to detect the degree of degradation of Pex14-GFP and β-actin was used as an internal control. (B) The calculated proportion of free GFP in the total band signal. The grayscale value of the strip was measured using ImageJ software. Error bars on the histograms represent the standard error of three repeated tests. Different letters on the bars for each treatment indicate significant difference at $p < 0.05$ based on Duncan's multiple range test.
